# P25 A complex case of mixed systemic sclerosis and myositis with severe multi-system involvement

**DOI:** 10.1093/rap/rkac067.025

**Published:** 2022-09-28

**Authors:** Sarah Fordham, Eleana Ntatsaki

**Affiliations:** Ipswich Hospital, Suffolk, United Kingdom; Ipswich Hospital, Suffolk, United Kingdom

## Abstract

**Introduction/Background:**

This case highlights the extent of multi-organ involvement that can be seen with scleroderma, or in our patients’ case, a scleroderma myositis overlap. It presents many diagnostic and management challenges and highlights the need for multi-disciplinary team involvement of these patients.

The nature of the cardiac involvement is rare and has required extensive input from the cardiologists. His gastrointestinal symptoms have been very troubling and are still not fully controlled. The Raynaud’s continues to be problematic, but treatment is confounded by his ongoing cardiac issues. He has tried multiple DMARDs and is currently receiving Rituximab.

**Description/Method:**

We present the case of a 63 year old gentleman with a diagnosis of diffuse systemic sclerosis with overlap myositis. He presented in 2016 with arthralgia, myalgia, sclerodactyly and Raynaud’s with digital ulceration. He is positive for Mi-2b and PM-Scl-75 antibodies. He was initially treated with methotrexate (oral and subcutaneous) and high dose prednisolone.

In 2019 he developed dyspnoea and bradycardia. Cardiac MRI suggested underlying scleroderma with non-ischaemic dilated cardiomyopathy. He also developed atrioventricular block, and required implantation of a cardiac resynchronisation device and defibrillator. Left ventricular ejection fraction has consequently improved from 30-35% to 40-45%. Pulmonary artery pressures are normal.

His scleroderma and muscle weakness were also deteriorating and he was subsequently given six pulses of IV cyclophosphamide followed by mycophenolate mofetil for maintenance. A muscle biopsy in 2019 confirmed necrotising myopathy with chronic neurogenic change.

He also noted worsening dysphagia, heartburn and bloating and was admitted in 2020 with aspiration pneumonia. OGD and colonoscopy proved gut dysmotility and small intestinal bowel overgrowth was suspected. These symptoms improved significantly whilst he was an in-patient on co-amoxiclav. Gastroenterology advised regular Rifaximin and Omeprazole. He also has mild basal interstitial lung involvement.

He was reviewed remotely by the Royal Free in July 2020.

In August 2020 he had two pulses of Rituximab for a flare of myositis. This was repeated in 2021. His creatinine kinase has normalised and he has responded well. His most recent Modified Rodnan Skin Score is 9 and muscle power 4+/5 in the proximal muscle groups. His skin, myositis and gastrointestinal symptoms have all improved.

Ongoing symptoms include dyspnoea for which he is awaiting an up to date ECHO and troubling gastrointestinal symptoms with recent video fluoroscopy showing moderate oropharyngeal dysphagia and a barium swallow showing mild to moderate aspiration with appearances of significant oesophageal dysmotility.

**Discussion/Results:**

Overall, the combination of symptoms present with the mixed scleroderma and myositis picture makes this an interesting case. The presence of two antibodies and the truly mixed picture that the patient presents has provoked many management challenges but has perhaps resulted in a wider variety of treatments for instance being able to offer Rituximab. Our team has only recently been involved in the patients care given a recent move to the area and so only very recent management decisions have been made by us with the guidance of the Royal Free.

The transfer of care for these patients can be challenging given the often complex histories and previous treatments and it has highlighted that we need extra time to assess patients such as these in our clinics especially when first getting familiar with them. Trying to engage all relevant specialities in a timely manner particularly more recently over the pandemic has proven to sometimes be difficult. As rheumatologists we often find ourselves overseeing many treatments and guiding patients to other specialities.

This case highlights many points for discussion. The severe multi-organ involvement has been very difficult to manage. The cardiac involvement has been serious and warranted pacing and resynchronisation therapy. Breathlessness continues to be an issue for the patient. Whilst the cardiologists are managing his cardiac failure medically there have been issues regarding the co-prescription of Entresto and Sildenafil as the patient suffers with hypotension. Bisoprolol clearly caused a worsening of his Raynaud’s and had to be switched. It is hard to know how to balance these issues going forwards.

The oesophageal dysmotility is of concern and given he has already had one serious admission with aspiration pneumonia is perhaps the most disconcerting.

**Key learning points/Conclusion:**

I believe the overarching learning point from this case relates to the importance of the MDT. The increasingly complex nature of connective tissue disorders with the potential to involve almost any organ system means as rheumatologists we must always be very open minded to patients developing new symptoms and ensure early referral to the appropriate speciality.

The second main learning point, which leads on from the first, is that not all treatments recommended by various specialities complement each other and it can be a very difficult balancing act to achieve the best outcomes for the patient.

The third is that over time a range of DMARDs will often be required to treat these patients who require multiple treatments and are very prone to relapse. Constant monitoring and easy access to services is essential to the good care of these patients. This has not always been easy over the pandemic.

I would like to discuss further management options for the gastrointestinal involvement which seems to impact greatly on the patients’ quality of life. It is, however, not only this which is concerning. Given the patients risk of aspiration, with his prior hospital admission and high level of immunosuppression, could his oropharyngeal dysphagia be the most concerning manifestation to date?

